# Small-scale metapopulation structure of a limnophilic fish species in a natural river system investigated using microsatellite genotyping by amplicon sequencing (SSR-GBAS)

**DOI:** 10.1186/s12862-023-02192-0

**Published:** 2024-01-02

**Authors:** Paul Meulenbroek, Manuel Curto, Paria Priglinger, Kurt Pinter, Spase Shumka, Wolfram Graf, Fritz Schiemer, Harald Meimberg

**Affiliations:** 1 Christian Doppler Laboratory for Meta Ecosystem Dynamics in Riverine Landscapes, Institute of Hydrobiology and Aquatic Ecosystem Management, Department of Water, Atmosphere and Environment, University of Natural Resources and Life Sciences, Vienna, Austria; 2 Institute for Integrative Nature Conservation Research, Department of Integrative Biology and Biodiversity Research, University of Natural Resources and Life Sciences, Vienna, Austria; 3 BIOPOLIS Program in Genomics, Biodiversity and Land Planning, CIBIO Centro de Investigação em Biodiversidade e Recursos Genéticos, Campus de Vairão, Vairão, Portugal; 4 Institute of Hydrobiology and Aquatic Ecosystem Management, Department of Water, Atmosphere and Environment, University of Natural Resources and Life Sciences, Vienna, Austria; 5 Faculty of Biotechnology and Food, Agricultural University of Tirana, Tirana, Albania; 6 Department of Limnology and Bio-Oceanography, University of Vienna, Vienna, Austria

**Keywords:** Genetic diversity, Habitat specialist, *Pelasgus thesproticus*, Population structure, Source-sink dynamic, Vjosa

## Abstract

Habitat niches of fish species can exert a strong influence on population structure, even on a small geographical scale. In this scope, *Pelasgus thesproticus* is a great model species to study connectivity in riverine environments owing to its naturally patchy habitat distribution. Furthermore, it is important to conduct such studies in near-natural systems to avoid the impact of human disturbances on the river, such as fragmentation, morphological changes and habitat degradation. In this sense, the Vjosa in Albania is an excellent study area. A total of 204 individuals were sampled from five locations in the lower Vjosa and two tributaries and genotyped with 33 newly designed microsatellites loci using high throughput sequencing. The application of microsatellite genotyping by sequencing revealed genetic structure and some differentiation, even at a small spatial scale (< 65 river km). A total of 500 alleles were found with an average of 0.93 private alleles among sites with rather low F_ST_ values (< 0.04). The extent of admixture observed in some populations indicate that the genetic structure is mainly influenced by upstream populations, either from the main river itself or from tributaries. In addition, the connection between a tributary and the other sites is disrupted by the flow regime, which is reflected in a high degree of divergence from the other populations. Our results indicate that hydrological conditions of the flowing river present strong barriers to gene flow, particularly in the upstream direction, but at the same time act as dispersal corridors in the downstream direction and exhibit source-sink dynamics in which upstream populations contribute disproportionately to downstream populations for this habitat specialist along the river. It is suggested that processes of colonization and reinforcement may play an important role in shaping the genetic structure of patchily distributed fish species in natural river systems. Future studies should increase the knowledge of dispersal factors, habitat heterogeneity, consequence of source-sink dynamics, and gene flow within the system, which will help to understand and maintain important processes related to metapopulation theory and the potential evolutionary consequences of habitat loss and fragmentation.

**Supplementary Information:**

The online version contains supplementary material available at 10.1186/s12862-023-02192-0.

## Introduction

Habitat isolation and the extent of movement of organisms across the landscape and functional connectivity are critical to the long-term existence of metapopulations, as the dispersal of individuals between subpopulations facilitates recolonization after local extinctions [1, 2]. Furthermore, migration and dispersal are fundamental components of metapopulation biology, linking a number of local populations to compensate for local extinction and enable the regional survival of a species, facilitating genetic resilience, demographic stability, and range expansion into unexploited habitats [3]. The life history and dispersal characteristics of a species have an influence on, and most likely interact with the distribution of the population in the river network. Many of the attempts to study the population connectivity and genetic structure of riverine fish within one river system have either focused on migratory fish [e.g. 4, 5], and/or those in highly fragmented river systems [e.g. 6, 7]. There are very few studies on fish species specialized in specific habitats distributed along a river, and these showed a lack of genetic structure within the river (e.g. [8]). Understanding the role of connectivity in riverine fish ecology (including the spatial structure of stream networks, force and direction of flow, scale-dependence of connectivity, shifting boundaries, complexity of behavior, and life histories) remains a challenge [9]. Various models have been proposed that predict how populations of taxa with different life-history traits and dispersal capabilities interact within riverine landscapes (see [10] and references therein). Fish species show preferences for certain water flow conditions in their habitat [11]. Thus, limnophilic species are restricted to stagnant waters, which occur only in patches in natural river systems. Little is known about how their genetic structure and metapopulation connectivity are shaped by natural habitat partitioning.

Natural systems in unimpaired hydromorphological states are rare in Europe. However, the Vjosa River in southern Albania is one example of a few remaining reference sites for dynamic floodplains [13]. Heterogenic habitat configurations provide conditions for a variety of ecological guilds. 31–33 fish species are inhabiting the Vjosa River system [14], while during the latest sampling [15], there were 18 species with spatial differences and high abundances of migratory species, such as the European eel [16]. Another study [17] provided the first insights into local fish habitat use, exhibiting distinct fish assemblages of different aquatic habitat types. Based on this and following the metapopulation concept, we chose *Pelasgus thesproticus* to study connectivity in riverine fish populations as a model species because it naturally has a patchy habitat distribution in this system. It is found in springs, ponds, backwaters, and ditches, usually in shallow, quiet water with dense vegetation, and they are able to survive summer droughts in pools remaining in river beds, springs, and wells [18]. No specimens were present in the main arm of the Vjosa River [17] and therefore higher flow may present strong barriers to gene flow. Previous studies for this genus have only focused on analysis of populations for different drainage systems [19] or on the evolutionary history [20].

Microsatellites play an important role in population genetics research, although the high cost of development and genotyping is a technical constraint and remains a major bottleneck. The use of microsatellite genotyping by amplicon sequencing (SSR-GBAS) utilizing amplicon sequences for allele calling based on Illumina technology allows the simultaneous screening of a higher number of markers and automation of data analysis, making the genotyping process less time and labor consuming. Further it allows the recovery of a higher number of alleles, and it is less prone to homoplasy since SNPs in the flanking regions of the repetition motive are considered. Genotyping can also be replicated more reliably, thereby removing biases that might prevent the concatenation of datasets from different analyses [21]. The entire sequence information for the allele call, the length information, and the SNPs are taken into account, resulting in a higher number of alleles than in traditional chromatogram-based microsatellite analysis, and even minor genetic differentiations that would otherwise go unnoticed can be detected [21–23].

In this study, we used molecular markers to investigate the population genetics of *P. thesproticus* by characterizing their neutral genetic diversity, structure, and population differentiation. We hypothesize that the shape of the branching network has an influence on metapopulation structure (the dendritic network approach; [12]), and expect that the hydrological conditions of the flowing river present strong barriers to gene flow, particularly in the upstream direction, but at the same time act as dispersal corridors in the downstream direction and exhibit source-sink dynamics in which upstream populations contribute disproportionately to downstream populations.

## Methods

**Fig. 1:**
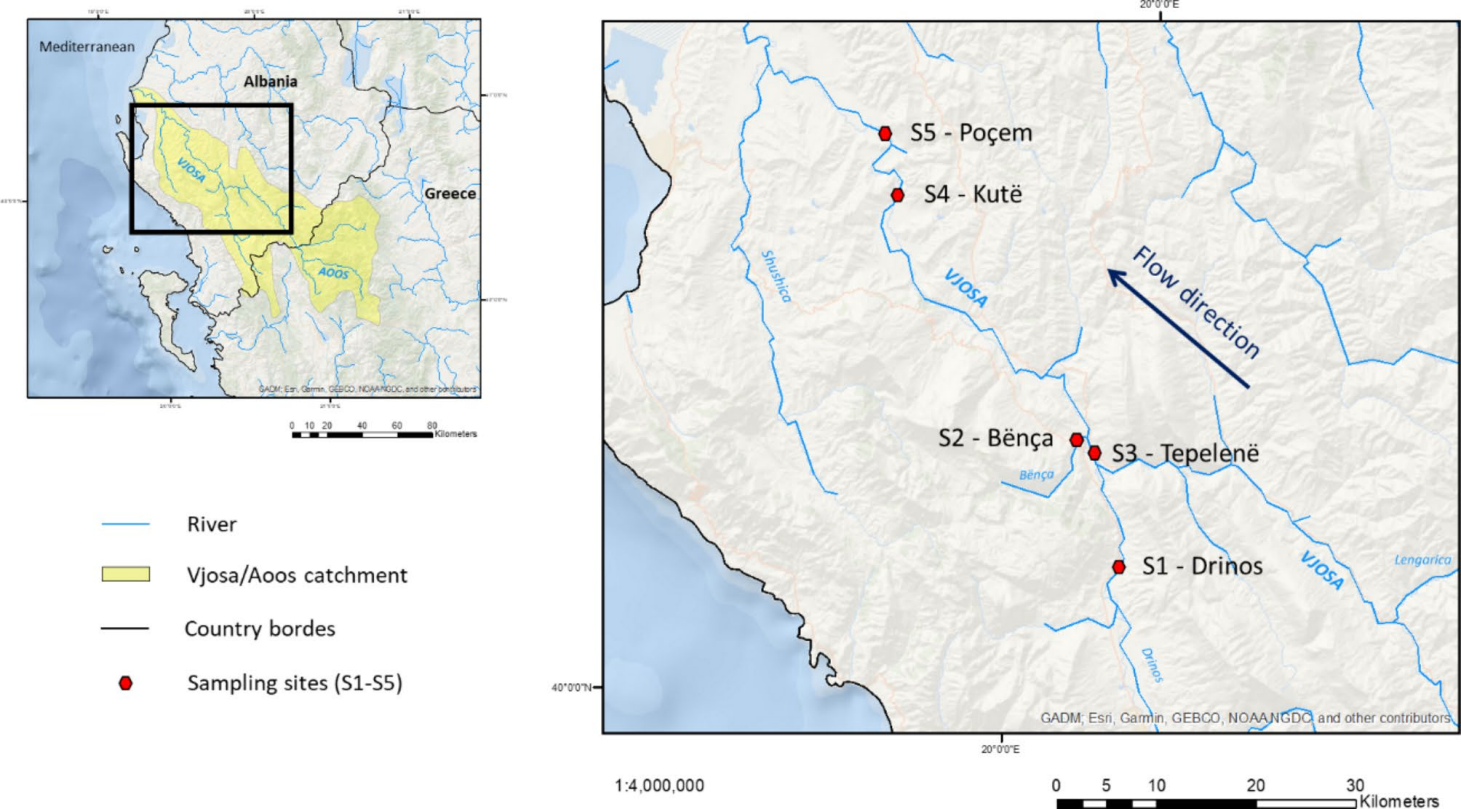
Catchment of the River Vjosa/Aoos, its main tributaries, and all sampling sites. The map is based on J Aguilar-Manjarrez [25] and B Lehner, K Verdin and A Jarvis [26]

This study was conducted in the Vjosa River catchment in Albania. Along its course, a variety of channel types occur, with gorges in the upper parts, braiding and anabranching sections in the middle and lower courses, and meandering stretches close to the river mouth. A detailed description of Vjosa and its accompanying landscape is available [24]. Including all permanently wetted tributaries, the entire river network provides approximately 1060 of unfragmented riverine habitats [16]. During several field visits, five sites inhabited by *P. thesproticus* were identified; three sites in the Vjosa River (Tepelenë, Kutë, and Poçem) and two tributaries, the Bënça and Drinos Rivers (Fig. 1).

The most upstream sampling point [S1 – Drinos (40°11’38.5” N, 20°05’03.3” E)] was located in a tributary, 12 km upstream of the confluence with the Vjosa, in the Drinos River, from which 31 specimens were sampled in a groundwater-fed ditch on a gravel bank.

The first sampling site in the main river (S3 – Tepelenë (40°17‘25.0” N, 20°01‘31.7” E)), was located 15 km downstream, with a spring present within the active channel and a sample size of 44 specimens. Two kilometers downstream joins the Bënça River.

The site S2 – Bënça (40°17’45.2” N, 20°00’34.2” E), wherein 55 specimens were sampled, is located 2 km upstream of the Vjosa River with a relatively high slope.

Site S4 – Kutë (40°28‘14.5” N, 19°45‘15.0” E), located 38 km downstream, is a small spring located on the right side within the active channel of the Vjosa and had a sample size of 45 specimens. 10 km further downstream S5 was located – Poçem (40°31‘27.2” N, 19°43‘58.2” E), from where 29 specimens of fish were sampled in a groundwater-fed ditch on a gravel bank of the main channel. The maximum distance between S1 – Drinos and S5 – Poçem is 63.5 km. In total, 204 specimens of *P. thesproticus* were sampled from September 2017 to May 2018, employing either a short shore seine or electrofishing. Fresh tissue samples were taken from the pectoral fins and directly preserved in 97% ethanol. All fish were released immediately afterwards.

Genetic variation of *P. thesproticus* populations was screened with microsatellite genotyping by amplicon sequencing (SSR-GBAS) utilising amplicon sequences for allele calling based on Illumina technology. The method takes into account the entire sequence information for the allele call, the length information, and the SNPs using the SSR-GBS pipeline (https://github.com/mcurto/SSR-GBS-pipeline). More specifically the alleles were defined in three steps: 1st allele length counts were used to define the genotype in a similar fashion of traditional microsatellite analysis; 2nd the corresponding reads were extracted and used to define SNP variants per length; 3rd each allele was defined as a unique sequence resulting into genotypes using the whole sequence information. As a result, the number of alleles is higher than in traditional chromatogram-based microsatellite analysis, and even minor genetic differentiations that would otherwise go unnoticed can be detected [21–23]. In the present study, we utilized simple sequence repeats (SSRs) to typify *P. thesproticus* in the Vjosa using next-generation sequencing. DNA extraction, SSR discovery, primer design for amplicon sequencing, SSR primer testing, PCR multiplex and Illumina MiSeq sequencing, sequence analysis, and SSR‑GBAS genotyping was done as described in [21]. All sequence data produced were deposited in the Sequence Read Archive (SRA) of GenBank under the project number PRJNA860787. Markers failed to genotype more than 40% of the individuals were excluded. Deviation from Hardy Weinberg Equilibrium (HWE) per loci, per population, were estimated using GenAlEx v6.503 [27]. The proportion of null alleles was estimated with the program FreeNA [28]. Markers showing a proportion of null alleles above 0.1 and deviating from HWE for all populations were deleted.

To have a comprehensive view on the genetic diversity patterns, several statistics were calculated using GenAlEx v6.503 [27]. Both the average number of alleles per locus (Na) and the effective number of alleles (Ae) were considered. While Na is correlated with genetic diversity, this value may be inflated by rare alleles. Ae is the number of equally frequent alleles necessary to achieve the expected heterozygosity making it a measure less sensible to biases created by a high number of rare alleles [29]. These values of diversity were complemented by estimates of observed heterozygosity (Ho), unbiased expected heterozygosity (uHe), and Shannon’s information index (I). The same program was used to estimate the mean number of private alleles (Np) per populations and F-statistics (F_ST_) as measures of genetic differentiation among populations. Divergence in allele frequency between populations based on Nei’s genetic distances [30] was visualized by building a UPGMA dendrogram as implemented in Populations v.1.2.32 [31] and a neighbor network using SplitsTree4 [32]. Population structure based on similarity among individuals was first evaluated through a principal Coordinate Analysis (PCoA) calculated with GenAlEx v.6.503 [27]. Population structure was further examined by assigning individuals to populations based on the Bayesian clustering method using STRUCTURE v2.3.4 [33, 34] as described by [23]. STRUCTURE ran for 100,000 generations followed a period of burning (100,000 generations) for K values between 1 and 10 (10 replicates each). Allele frequencies were considered to be correlated and the admixture model was used. The optimal K value was chosen using delta K (∆*K*) statistic as implemented in STRUCTURE HARVESTER (http://taylor0.biology.ucla.edu/structureHarvester/) [35].

## Results

Of the 1,161,779 paired reads generated for primer design, 19,689 contained SSR motifs and complied with our quality criteria. We generated a total number of 9,004,896 paired reads, of which 8,149,965 passed our quality control procedure, yielding 1731 to 98,774 sequences per sample. In total, 48 primer pairs were designed, of which 41 primers amplified loci with less than 40% of missing data. Eight loci either showed HWE deviations for all populations or resulted in a proportion of null alleles above 0.1 and were excluded. This resulted in a final dataset of 33 markers (primer sequence, repetition motif and amplicon length are presented in Supplementary Tables 1 and their genetic diversity statistics are shown in Supplementary Table 2.).

A total of 500 alleles were found in the studied samples of *P. thesproticus* across the 33 loci. The mean number of alleles per locus ranged from 3 to 37 with an average of 15.2. Among the 5 populations investigated, the highest number of private alleles was observed in Bënça (1.27). The genetic diversity indices were comparable for all sites, with the highest values observed in Tepelenë (Table 1). Observed and expected heterozygosity differed by less than 0.08 for all sites. The most downstream sampling site showed the smallest number of alleles per locus (7.58 ± 0.64). A significant overall F_ST_ value (*p* < 0.01) of 0.04 was obtained with a 95% confidence interval ranging between 0.03 and 0.04. Pairwise genetic differentiation among the five populations showed low F_ST_ values (< 0.04), indicating low differentiation. The highest F_ST_ values were found when comparing all other populations with Bënça. However, the lowest values were found in the populations of Drinos and Tepelenë (0.007). Only for Bënça most of the loci did not deviate from the Hardy-Weinberg equilibrium (Table 1).

**Table 1:** Genetic diversity indices (values are given as mean ± SE) and the pairwise population F_ST_ values of 5 *Pelasgus thesproticus* populations derived from 33 microsatellite loci

	N	Na	Ae	Np	I	Ho	uHe	F	%dHWE	S2	S3	S4	S5	
S1 - Drinos	30.64 ± 0.14	9.66 ± 0.88	4.99 ± 0.56	1.03 ± 0.23	1.63 ± 0.13	0.63 ± 0.04	0.69 ± 0.04	0.07 ± 0.03	69.70	0.027*	0.007	0.019*	0.023*	S1 - Drinos
S2 - Bënça	52.64 ± 0.55	8.21 ± 0.77	3.76 ± 0.34	1.27 ± 0.31	1.42 ± 0.11	0.61 ± 0.04	0.65 ± 0.04	0.05 ± 0.02	33.33		0.024*	0.023*	0.038*	S2 - Bënça
S3 - Tepelenë	43.61 ± 0.14	10.15 ± 0.94	4.83 ± 0.51	1.03 ± 0.22	1.62 ± 0.12	0.64 ± 0.04	0.69 ± 0.04	0.05 ± 0.02	57.58			0.014*	0.026*	S3 - Tepelenë
S4 - Kutë	43.73 ± 0.50	9.58 ± 0.87	4.45 ± 0.46	0.7 ± 0.15	1.57 ± 0.11	0.63 ± 0.04	0.68 ± 0.04	0.05 ± 0.03	57.58				0.027*	S4 - Kutë
S5 - Poçem	28.24 ± 0.17	7.58 ± 0.64	3.98 ± 0.42	0.61 ± 0.14	1.42 ± 0.11	0.62 ± 0.04	0.65 ± 0.04	0.04 ± 0.03	72.72					S5 - Poçem
Average number of individuals per locus (N), average number of alleles per locus (Na), number of effective alleles (Ae), average number of private alleles (Np), Shannon’s information index (I), observed heterozygosity (Ho), unbiased expected heterozygosity (uHe), inbreeding coefficient (F), percentage of loci deviating from HWE (%dHWE).										Pairwise population F_ST_ values. Values significantly higher than 0 are marked with an asterisk (*).				

**Fig. 2:**
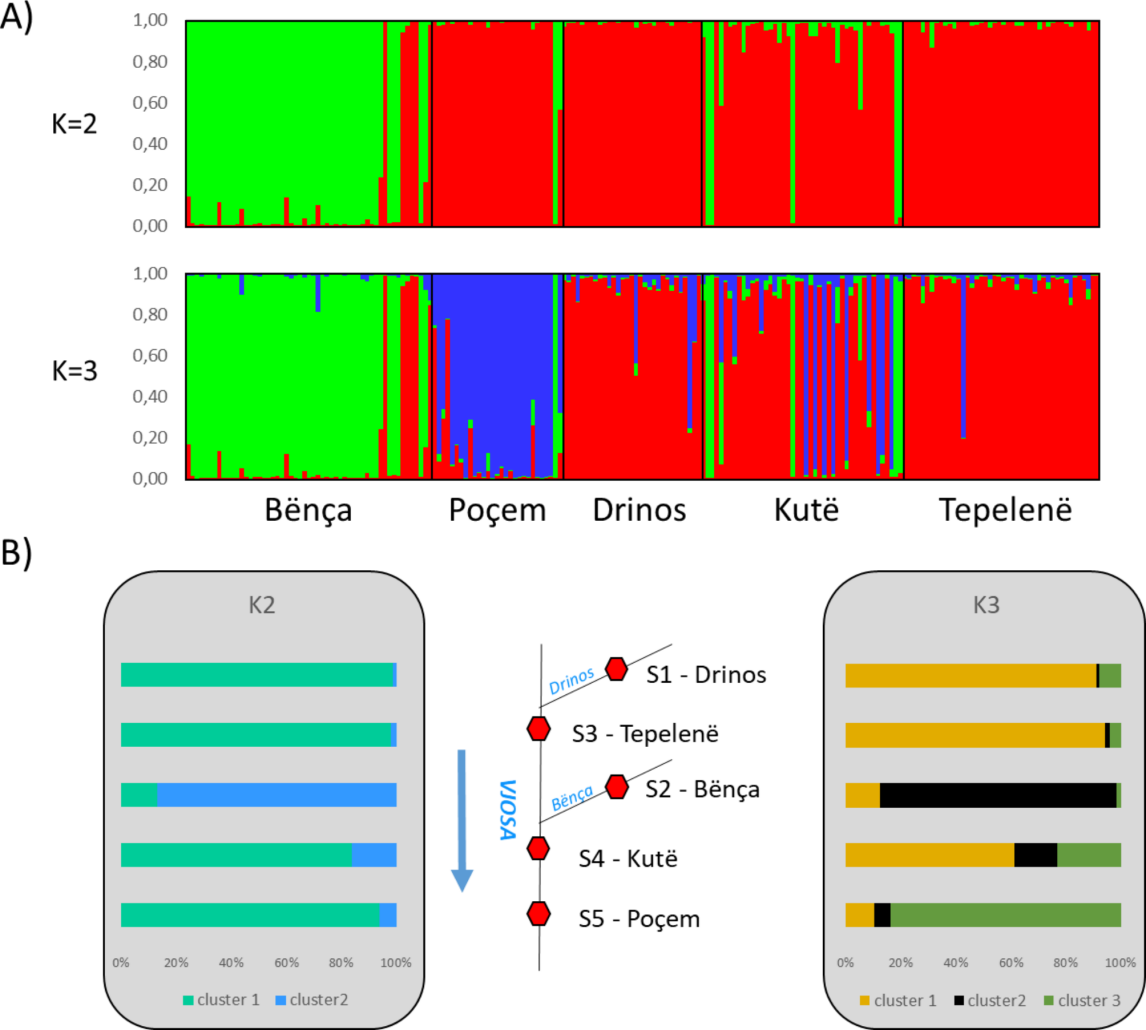
(**A**) STRUCTURE analysis (admixture model) for *Pelasgus thesproticus* (optimal cluster K = 2, suboptimal clusters K = 3) derived from 33 microsatellite loci. Each bar representing a single individual, and each color representing proportion of membership (population) with regard to each genetic cluster. (**B**) Average cluster assignment per population for K = 2 and K = 3

**Fig. 3:**
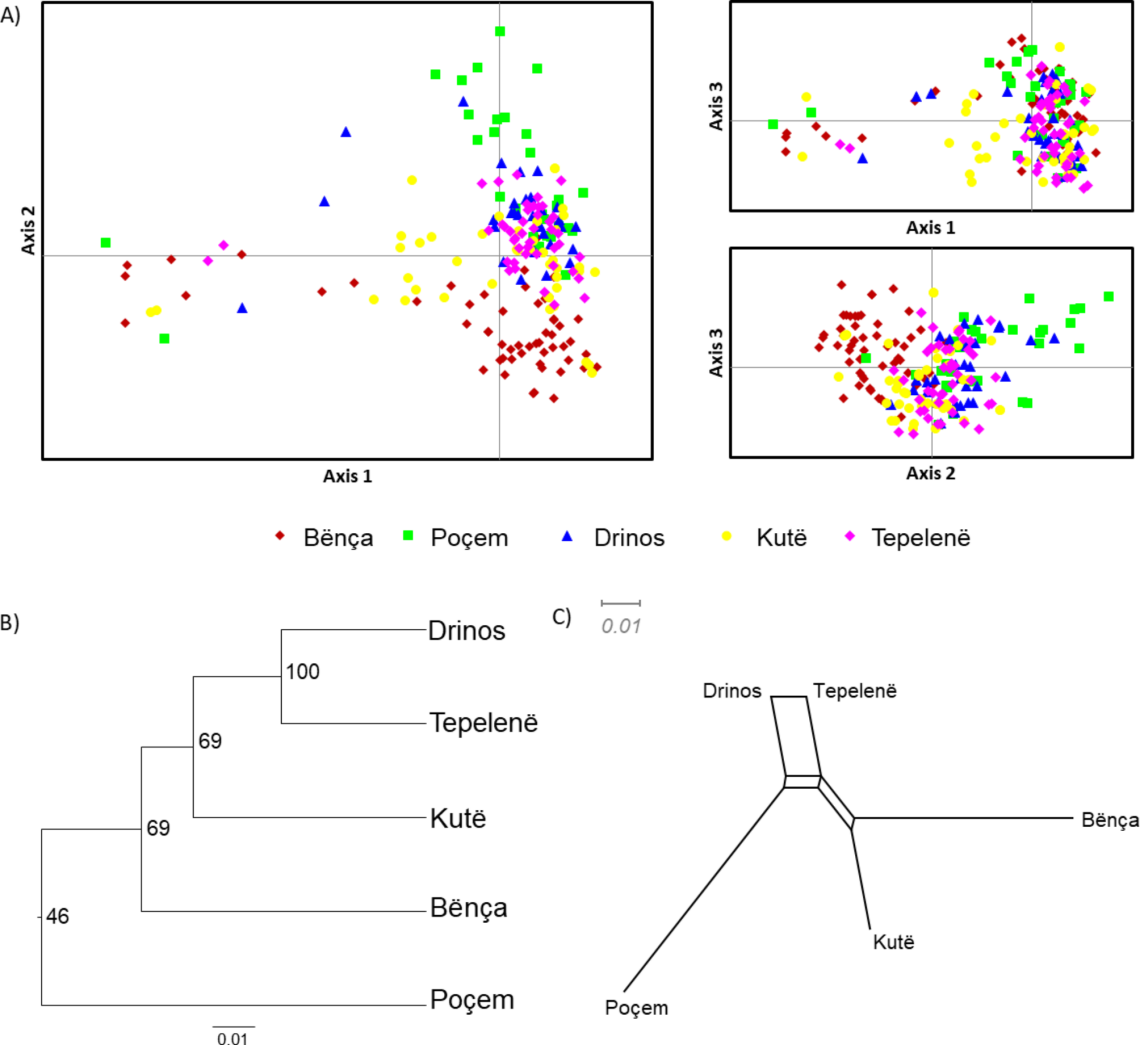
(**A**) Principal Coordinate Analysis (PCoA) plots to illustrate the genetic similarity of *Pelasgus thesproticus*. Nei’s genetic distance between populations: (**B**) UPGMA dendrogram with bootstrap support values, B: (**C**) Neighbor network

Based on the delta K (ΔK) values, K = 2 was considered the best fit for the data (ΔK = 259.91). The STRUCTURE HARVESTER results also suggested K = 3 as the second-best fit for the data (ΔK = 3.43). Individuals from Bënça were assigned to one cluster (green) and the remainder to the second (red). Some individuals showed mixed assignments to both clusters and were assigned to a different cluster than the other members of their population. For K = 3, most individuals from Poçem were assigned to a third cluster, with a certain proportion also found in Kutë (Fig. 2A). These results were also illustrated by averaging clustering assignment per population (Fig. 2B). For K = 2, 86.8% of Bënça was assigned to cluster 2, whereas the others range from 1.2 to 16.2%. For K = 3, Bënça stood out with 85.6% in Cluster 3 and Poçem with 83.7% in Cluster 2. Principal Coordinate Analysis (PCoA) explained 5.77%, 3.92% and 3.06% of the variation for axis 1 to 3 respectively. This analysis showed a similar segregation pattern for Bënça and Poçem (Fig. 3A). Both the UPGMA dendrogram (Fig. 3B) and neighbor network (Fig. 3C) showed Drinos and Tepelenë to be more genetically similar populations. Similar to the STRUCTURE analyses, Bënça and Poçem are the most divergent populations.

## Discussion

We were able to characterize genetic structure patterns of *P. thesproticus* on a relatively small geographical scale. Although this can be partially related to its ecology, it can also be explained by the high amount of information provided by the SSR-GBAS methodology. This method increases the ability of retrieving genetic variation information in two ways when compared with traditional microsatellite genotyping. First, it permits multiplexing a high number of markers with relatively low costs [22]. Our final dataset contained 33 markers which is more than the 10 to 20 that are usually implemented [36]. Second, because the whole sequence information is used, it was possible to define a high number of alleles per locus (up to 37), allowing to recover small differences between close related populations [22].

Small limnophilic fish species are likely to be strongly influenced by hydrological factors and the connectivity between populations might be strongly affected by the natural flow regime. For a species like *P. thesproticus* that was not located in the flowing conditions as main arm of the river, but rather in quiet water with dense vegetation (e.g. springs, ponds, backwaters, and ditches), we can hypothesize that there is downstream connectivity but less opportunity for upstream migration, which should be reflected in the genetic structure of the species. So far, we included five populations of the Vjosa River system, which is one of the last unregulated rivers in Europe. We showed that the genetic structure and connectivity at a small spatial scale (< 65 km) are shaped by natural habitat patchiness. Our results are consistent to some extent with the hypothesis of downstream connectivity between populations to explain genetic structure: The Drinos population is the most upstream and forms a cluster with Tepelenë, the neighboring downstream population. The population geographically closest to Tepelenë, Bënça, diverges most significantly from all other samples. Bënça is a tributary that differs from the mainstem in terms of hydrology and species composition [24]. It joins the Vjosa near Tepelenë, but apparently there is no exchange between Tepelenë and Bënça or it is not measurable. This strongly suggests that connectivity between the two streams is disrupted by the flowing regime, which is in accordance to the placement of Bënça as the second most divergent population in the UPGMA analysis and the fact that almost all of its individuals are assigned to an independent cluster across all K values in the structure analysis. This is also indicated by the extent of admixture observed in the population in Kutë. Only here there were individuals assigned to the main Bënça cluster. Together with the observation of high genetic similarity of Kutë to the most upstream populations indicates a major gene flow direction from upstream to downstream. In this context the high genetic differentiation between Poçem and the remaining upstream populations, especially the neighboring Kutë population, is surprising. The Poçem sampling site is located in the middle of a small, alluvium-fed pool in the middle of a branching section of the river that is inundated during the annual winter floods with rather a small total population. It is possible that this is an ephemeral population descended from individuals from an unsampled gene pool. Although this population has a relatively lower genetic diversity compared to the remaining populations, this difference is low. The lack of inbreeding (F = 0.04) indicates that the founder effect is minimal. Furthermore, this population also has the highest number of markers that deviate from the HWE, indicating a lack of panmixia. Alternative hypothesis could be that these individuals do not breed locally and result from the expansion from a nearby unsampled population or other historical colonization patterns. Genetic structure in the remaining populations is best explained by a source-sink dynamic in which upstream populations contribute disproportionately to downstream populations. In this case, Poçem is a population that might have been founded by an unknown upstream source. However, some upstream gene flow cannot be completely ruled out by our results since a few individuals from some populations (Bënça and Kutë) are assigned to major structure clusters found in downstream populations (Kutë and Poçem, respectively). These may have been favored in sporadic conditions when river flow is lower. For phytophilic species such as *P. thesproticus*, upstream transport of the sticky fish eggs by waterfowl is also imaginable. Regardless if the observed pattern is explained by such source sink dynamics or by the interplay between downstream and upstream migration likelihood, our data show that metapopulation dynamics processes of colonization and reinforcement might play a significant role in shaping the genetic structure of patchy distributed fish species in natural river systems. The low difference in genetic diversity among populations supports the idea that these dynamics have been relatively stable. Disturbing it might also constitute a risk for such species, when recolonization of habitat patches is impeded or important source populations are impacted.

Based on the genetic structure patterns of *P. thesproticus* we can conclude that river flow dynamics and the position in a dendritic network play an important role in shaping connectivity among limnophilic fish populations. Areas of stronger water flow work as gene flow barriers and dispersal corridors in the upstream and downstream directions, respectively. The flow seasonality of Mediterranean rivers coupled with the fact that these fish species tend to occupy slow water secondary arms results in intricate metapopulations dynamics where geographically close populations may be genetically divergent. This results in a patchy structure pattern that should be considered when monitoring limnophilic fish genetic diversity. Information on the hydromorphological characteristics of the respective river basins and migration on different scales may further be used as a basis for more detailed investigations. Environmental differences, habitat stability, lateral and longitudinal hydrological connectivity, distance, gradient, and other landscape factors will be considered in ongoing studies (riverine landscape genetics; (see [37]) and references therein). Future work will include additional populations to support this picture. Preliminary analysis of gene flow using BayAss suggests connectivity among main corridor populations in our dataset, but has been inconclusive as to the direction of migration. Ongoing surveys will specifically address these investigations. We expect that the inclusion of additional metapopulations and the inclusion of mtDNA data will reveal new pathways of connectivity between the populations as an example of the complex metapopulation structure some fish species form in natural river systems. The Vjosa is an ideal laboratory for studies of single and multiple species metapopulation dynamics, with the potential to test and advance fundamental concepts and demonstrate how dispersal factors, habitat heterogeneity, and gene flow maintain important processes related to metapopulation theory and the potential evolutionary consequences of habitat loss and fragmentation.

### Electronic supplementary material

Below is the link to the electronic supplementary material.

Supplementary Material 1

## Data Availability

All sequence data produced were deposited in the Sequence Read Archive (SRA) of GenBank under project number PRJNA860787.
