# Supplementary material for: Small-scale metapopulation structure of a limnophilic fish species in a natural river system investigated using microsatellite genotyping by amplicon sequencing (SSR-GBAS)
Source: BMC Ecol Evol. 2024 Jan 2;24:1. doi: 10.1186/s12862-023-02192-0 (PMC10759740; doi:10.1186/s12862-023-02192-0)
Supplement: Supplementary file 1 — Supplementary Material 1 [file 12862_2023_2192_MOESM1_ESM.docx]

Supplementary Table 1. Information of the final set of 41 SSR markers, concerning primer sequence, repetition motif and amplicon length. Marker names with asterisk were deleted due to HWE deviations and presence of null alleles.

| LOCI | Motif | No. of repeats | Forward | Reverse | Amplicon Length |
| --- | --- | --- | --- | --- | --- |
| PE1 | TATC | 19 | ACATGACCTTTAGCGAATGA | CCCACAGGAAAAGACAATCA | 440-524 |
| PE2 | ATCT | 17 | TTACTTGAATGGGTTTATTTGC | AGGATTATGATTTTGTTGAGGG | 326-462 |
| PE3 | CTTCT | 14 | CACGTGGTTGTTTTTCATGT | TCGAATAGAGTCGAATAGAGTC | 371-426 |
| PE4* | TCAA | 10 | TTTGGTTGGACTATCCTCAC | AATCATCAACCGTTTCAATTGT | 398-414 |
| PE5 | GAATA | 9 | ACCCTATGAGAAAGGAAACG | CAACCACGATTCAAACTCAG | 411-481 |
| PE6 | AGAT | 10 | TGTTTGTCACAACACATTTTG | CAAGTGCTGACAATGCATTA | 405-590 |
| PE9 | CAAC | 7 | CTTGTGTCATCCCTTTAGGT | CGGTCTGTTATCGTAGGTAC | 416-520 |
| PE10 | ATGG | 7 | TACTCACAAAGCCACGTTTA | ACAAGAAGCCCAAATCTGAT | 457 |
| PE11 | ATGA | 8 | TCACACACTTCTACGATGTT | TAGACAACTTCTACCCGACA | 424-444 |
| PE12 | ATCT | 12 | TCATAAACTTCAGCGTTTGG | ACATGTTGTGTTCCTCATGA | 408-480 |
| PE13 | GTTTA | 7 | TGTCTCAGTTGAAATAAGAGAG | TACAGCATCTGACACACATC | 405-420 |
| PE14 | AGAT | 8 | CCGTCATCAGCTTTAATGTG | TTACCATGCAAGTGACTCTC | 463-487 |
| PE15 | AGGAT | 7 | TGGGCAGGATAGGATATGAT | CACATGTTTACTTTGCCTCC | 424-451 |
| PE16* | TATC | 23 | AGCTGCGATGTCTATAAAGG | TGACAAGCTCAACAACATGA | 423-507 |
| PE17* | TTTA | 11 | GCAACATGCTTATGAGATGG | GTGACCACTAGATGTTGCTA | 333 |
| PE18 | TTCT | 9 | AGCTTCCACGTCCTCTATAT | TTTAATCCCTTTCCTTTTGCC | 396-504 |
| PE19 | TATG | 7 | GATGAGATCTGCCACGATG | ATCTTCCCTTCCACTAAAGC | 424-472 |
| PE20 | ATCT | 12 | ATAATTGGCACATTGTGGTG | TCCTGATGTACTTGCAAACT | 427-487 |
| PE22 | CATT | 7 | CTCAGTAATGCTCGCTGAAT | AGTATTTGCAATAATGGCGC | 408-428 |
| PE23 | GGAT | 17 | TAGATTTGTTGAGTGCTGGG | ACTAGCTGTTGCCAATGTAA | 421-473 |
| PE24 | GATT | 11 | TGACTTTCTAAGTGGCATCC | TGCATGACACATTAGATCAGA | 418-430 |
| PE25* | ATCT | 8 | CCACTAATGGTCTTACTGGG | CAATTGAGTTTGGTGCCATT | 483-539 |
| PE26 | AGAA | 15 | ACAGTCAACATCAACCTGTT | ATGCCTATTGCCTATTGAAAT | 417-454 |
| PE28 | AATTG | 6 | TTTCTGCACGGAGACTTTAG | TTATTGTTATCTGCCTGGCC | 432-442 |
| PE29 | ATTGC | 7 | TCAAGGTATCTAACGCTGTG | GTTCCAGGATCATTGGCTAA | 414-444 |
| PE30* | TCTA | 16 | TCCATGTGCCATCTGAAATT | AAGAGAAGCCTGCTGTTTT | 407-547 |
| PE31 | CTATT | 17 | ATGCATAATGGGTTGTAGCT | CTTCTATGTCAGTGCTGTGT | 412-472 |
| PE34 | TCTG | 10 | ACTTAGTTTCCATCGCGTT | GACTCATCCTTTTGAACAGC | 428-444 |
| PE35 | TTCA | 10 | AATGTTATTAGTTTTGGCAGGA | CCATGATGCCCTCTGTTTAG | 356-482 |
| PE36* | GATA | 8 | CACCTCCAGATTTATCCCAG | TATTTACTGGCTGGGTTCTC | 456-517 |
| PE37 | TATC | 12 | GTTGTCAATAATCTGCCATCA | TTAATTTTTGCAGGCATGGG | 399-461 |
| PE38* | ATAG | 17 | TGTGGGAAGTGAAGTTTTCT | TCATTTTCTCGTCTTGACCA | 386-520 |
| PE39 | AGAT | 16 | TCGAATGCAGAACTACACAA | GGGGGTTTATTTAGCGTAGT | 401-504 |
| PE40* | AGAAG | 7 | TGATAGAGAAGAGTGTCCTGA | GCCAAAGAAAATTGTGACTTC | 400-445 |
| PE41 | TTTC | 20 | ACATGAGAGTTTAAACATGGT | CAGAGAGCTGAGGAGACTG | 370-449 |
| PE42 | ATTCT | 10 | ACCTATAGCTGGTATGTGGT | TGCTCTGATGTCACTTTCAA | 410-440 |
| PE43 | AAGA | 9 | TAATTGCATCAGGGTTCACA | GTTTCAGAGAATGACCCAGT | 431-508 |
| PE44 | GATA | 12 | GGGTTCATGAGGCTTTGATA | AGGCTGATTCTTTTGCATTT | 417-493 |
| PE46 | TCTCT | 10 | ACAGCAGAGTGACTTTTGAA | ATAGATGCAACCAGTACAGG | 413-484 |
| PE47 | CTAT | 18 | GGTCACTGAAACAATCCGTA | CGAGAGGCTTAGTACTAGCT | 427-479 |
| PE48 | GTTT | 10 | ACTGAAACATGATTGGGAGT | GACTGGACTGGACATAACAG | 421-448 |

Supplementary Table 2. Statistics of the final set of 41 SSR markers for *Pelasgus thesproticus*. N= Sample size, Na=number of alleles, Ne= number of effective alleles, I= information index, Ho= observed heterozygosity, He= expected heterozygosity, uHe= unbiased expected heterozygosity, F= fixation index.

| LOCI | N | Na | Ne | I | Ho | He | uHe | F |
| --- | --- | --- | --- | --- | --- | --- | --- | --- |
| PE1 | 182 | 17 | 6.461 | 2.150 | 0.775 | 0.845 | 0.848 | 0.083 |
| PE2 | 202 | 17 | 8.246 | 2.400 | 0.629 | 0.879 | 0.881 | 0.285 |
| PE3 | 204 | 12 | 4.899 | 1.809 | 0.735 | 0.796 | 0.798 | 0.076 |
| PE4 | 204 | 12 | 4.856 | 1.809 | 0.623 | 0.794 | 0.796 | 0.216 |
| PE5 | 204 | 7 | 1.341 | 0.518 | 0.225 | 0.254 | 0.255 | 0.113 |
| PE6 | 201 | 22 | 3.656 | 1.792 | 0.582 | 0.726 | 0.728 | 0.199 |
| PE9 | 191 | 37 | 10.988 | 2.915 | 0.775 | 0.909 | 0.911 | 0.148 |
| PE10 | 176 | 3 | 1.810 | 0.665 | 0.426 | 0.448 | 0.449 | 0.048 |
| PE11 | 204 | 6 | 1.514 | 0.704 | 0.265 | 0.340 | 0.340 | 0.220 |
| PE12 | 193 | 19 | 12.271 | 2.670 | 0.865 | 0.919 | 0.921 | 0.058 |
| PE13 | 204 | 5 | 1.257 | 0.482 | 0.206 | 0.204 | 0.205 | -0.007 |
| PE14 | 203 | 7 | 3.317 | 1.449 | 0.601 | 0.699 | 0.700 | 0.140 |
| PE15 | 204 | 8 | 4.144 | 1.587 | 0.706 | 0.759 | 0.761 | 0.070 |
| PE16 | 152 | 54 | 21.684 | 3.410 | 0.612 | 0.954 | 0.957 | 0.359 |
| PE17 | 202 | 7 | 2.774 | 1.183 | 0.356 | 0.640 | 0.641 | 0.443 |
| PE18 | 204 | 9 | 1.811 | 1.048 | 0.426 | 0.448 | 0.449 | 0.048 |
| PE19 | 204 | 18 | 3.925 | 1.923 | 0.657 | 0.745 | 0.747 | 0.119 |
| PE20 | 187 | 19 | 8.949 | 2.440 | 0.775 | 0.888 | 0.891 | 0.127 |
| PE22 | 204 | 9 | 2.924 | 1.283 | 0.721 | 0.658 | 0.660 | -0.095 |
| PE23 | 194 | 21 | 7.928 | 2.445 | 0.758 | 0.874 | 0.876 | 0.133 |
| PE24 | 204 | 4 | 2.075 | 0.947 | 0.431 | 0.518 | 0.519 | 0.167 |
| PE25 | 204 | 20 | 3.790 | 1.744 | 0.564 | 0.736 | 0.738 | 0.234 |
| PE26 | 202 | 19 | 5.601 | 2.086 | 0.807 | 0.821 | 0.824 | 0.018 |
| PE28 | 204 | 4 | 1.267 | 0.413 | 0.216 | 0.211 | 0.211 | -0.024 |
| PE29 | 204 | 10 | 2.135 | 1.066 | 0.672 | 0.532 | 0.533 | -0.263 |
| PE30 | 200 | 35 | 14.583 | 2.984 | 0.535 | 0.931 | 0.934 | 0.426 |
| PE31 | 204 | 15 | 5.684 | 2.061 | 0.828 | 0.824 | 0.826 | -0.005 |
| PE34 | 204 | 6 | 2.705 | 1.153 | 0.598 | 0.630 | 0.632 | 0.051 |
| PE35 | 182 | 23 | 1.993 | 1.365 | 0.445 | 0.498 | 0.500 | 0.107 |
| PE36 | 202 | 13 | 2.173 | 1.149 | 0.465 | 0.540 | 0.541 | 0.138 |
| PE37 | 204 | 28 | 7.448 | 2.428 | 0.770 | 0.866 | 0.868 | 0.111 |
| PE38 | 175 | 38 | 14.254 | 2.975 | 0.446 | 0.930 | 0.933 | 0.521 |
| PE39 | 199 | 26 | 6.413 | 2.290 | 0.774 | 0.844 | 0.846 | 0.083 |
| PE40 | 189 | 4 | 1.669 | 0.744 | 0.143 | 0.401 | 0.402 | 0.644 |
| PE41 | 191 | 16 | 6.724 | 2.128 | 0.791 | 0.851 | 0.854 | 0.071 |
| PE42 | 204 | 6 | 2.247 | 0.955 | 0.471 | 0.555 | 0.556 | 0.152 |
| PE43 | 203 | 26 | 7.198 | 2.316 | 0.847 | 0.861 | 0.863 | 0.016 |
| PE44 | 195 | 31 | 10.565 | 2.690 | 0.846 | 0.905 | 0.908 | 0.065 |
| PE46 | 197 | 18 | 6.824 | 2.198 | 0.812 | 0.853 | 0.856 | 0.048 |
| PE47 | 204 | 21 | 7.018 | 2.305 | 0.819 | 0.858 | 0.860 | 0.045 |
| PE48 | 200 | 11 | 2.964 | 1.575 | 0.460 | 0.663 | 0.664 | 0.306 |
